# A Comparative Study of Tooth Shade Selection Methods Between Visual, Smartphone Camera, and Vita Easyshade Spectrophotometer

**DOI:** 10.7759/cureus.73338

**Published:** 2024-11-09

**Authors:** Rashed Alsahafi, Motaz Almaghraby, Eyad Almasri, Abdulrahman Banafa, Abdullah A Marghalani

**Affiliations:** 1 Department of Restorative Dental Sciences, College of Dentistry, Umm Al-Qura University, Makkah, SAU; 2 College of Dentistry, Umm Al-Qura University, Makkah, SAU; 3 Department of Preventive Dentistry, Umm Al-Qura University, Makkah, SAU

**Keywords:** aesthetics, dentistry, smartphone, spectrophotometer, tooth shade

## Abstract

Objectives: Recently, dental esthetics have increasingly emphasized appearance, with tooth shade selection complicated by the enamel and dentin’s translucency and opacity. Smartphone cameras are useful for shade matching. Currently, no study has directly compared the accuracy of this method. Therefore, the aim of this study is to compare the accuracy of the visual method, smartphone photography, and the Vita Easyshade spectrophotometer, particularly for chroma, value, and hue.

Methods: This cross-sectional study involved a convenient sample of 82 dental students from the College of Dental Medicine at Umm Al-Qura University, including those in their 5^th^, 6^th^, and 7^th^ (intern) years. The VITA classical shade guide was utilized to compare three methods of shade selection: the conventional visual method, smartphone photography, and the Vita Easyshade spectrophotometer. The percentage accuracy for each method was calculated with a 95% confidence interval (CI), using the visual method as the reference standard. Statistical comparisons of the shade selection methods, as well as gender differences, were performed using a Chi-Square test. Fisher’s exact test was applied where expected cell counts were below five. Stata software was used with a significance threshold of 0.05.

Results: The current study included 82 subjects; 42 were females and 40 were males. There was no significant difference between male and female students in the measured accuracy by using the three methods except for hue when the photo method was used (p=0.015). For chroma (p=0.094), value (p=0.965), and hue (p=0.094), the photo method was comparable in accuracy to the visual method. The accuracy was slightly higher for the photo method, but this difference was not statistically significant. The Easyshade was also comparable to the visual method in chroma (p=0.674), significantly lower when assessing value (p=0.002) and not statistically lower when assessing hue (p=0.094). Comparing the results of the same method, the accuracy of shade selection was highest for chroma, followed by value, and lowest for hue.

Conclusion: Within the limitations of this study, digital photography using a smartphone camera may serve as a reliable method for clinicians in shade selection during clinical practice.

## Introduction

Esthetics have become more significant for both dental professionals and patients in recent years. Previously, the primary focus in dental treatment was on functional requirements, but in today's context, there has been a shift towards dental esthetics [1].

It can be challenging for dental practitioners to select the appropriate tooth shade to accurately match the natural look of teeth [2], especially with the complexity of tooth structure. First, enamel is the hard, outermost layer of the tooth, consisting mainly of hydroxyapatite crystals. Enamel is formed from a complex, intricate structure composed of tightly packed rods or prisms, each approximately 5-8 micrometers in diameter. These prisms run from the inner dentin to the outer surface [3], arranged in a complex manner that reflects light in a special way according to the angle and direction of the prism [4]. Enamel is naturally translucent, allowing some light to pass through. This translucency gives teeth their characteristic appearance and allows the underlying dentin to influence the overall color. While enamel is primarily translucent, it also possesses varying degrees of opacity, contributing to the reflection and absorption of light, which affects the perceived color [5]. The natural hue of enamel typically ranges from white to light yellow. The underlying dentin, which has a yellowish tint, significantly influences this hue. Enamel color can vary among individuals and even among different teeth in the same mouth. These variations are influenced by factors such as genetics, age, and wear [6]. Second, dentin lies beneath the enamel and makes up the bulk of the tooth structure. It is less hard than enamel but more resilient, composed of microscopic tubules (dentinal tubules) [3]. The structure of dentin is highly dynamic, capable of producing secondary dentin in response to stimuli such as decay or wear, which make the teeth more yellowish and more opaque [7].

Additionally, when selecting the shade of the tooth, several factors come into play, including the transparency and opacity of teeth, variations in lighting conditions, eye fatigue effects, and color vision issues [2].

The VITA classical A1-D4® (Vita Zahnfabrik, Bad Säckingen, Germany) system was created using empirical data in 1956. It has been one of the most commonly used systems for decades and contains 16 shade tabs [8]. The tabs are organized into four hue groups formulated by letters: A for reddish-brown, B for reddish-yellow, C for grey, and D for reddish-grey [9]. A precise grouping of letters consists of shade tabs that share the same hue. Additionally, each hue group is organized further based on ascending chroma and descending value, following a numerical order like B1, B2, B3, and B4 [10].

Conventional visual tooth shade selection is a crucial aspect of cosmetic dentistry.

Dentists employ shade guides in controlled settings with standardized lighting to precisely match the natural tooth color of the patient. Different lighting sources, like daylight or artificial light, can impact color perception; hence, maintaining consistent lighting is crucial for dependable shade matching [11]. Despite being subjective and reliant on the clinician’s expertise, this process remains a cornerstone in cosmetic dentistry. Research indicates that experienced clinicians can achieve notable accuracy in shade matching through this method [12].

Spectrophotometers: A dental spectrophotometer is a specialized instrument used in dentistry to measure the color of teeth and dental materials accurately. This device helps dental professionals select the appropriate shade by measuring the intensity of the reflected light at different wavelengths. This data is then used to calculate the color parameters [5].

Several studies have compared various methods for shade selection, including visual and instrumental methods (such as spectrophotometers) [12]. In general, dental spectrophotometers provide the highest overall accuracy and precision if specific factors are controlled [12].

Utilizing a smartphone camera for digital tooth shade matching is becoming increasingly popular in dentistry due to the prevalence of mobile technology. This approach offers convenience and accessibility, rendering it suitable for specific shade-matching situations. In essence, employing a smartphone camera for this purpose presents a practical and economical alternative in certain clinical contexts. Although it may lack the precision of dedicated dental tools, it remains useful for initial shade evaluation, patient interaction, and fundamental shade choice [13].

To date, no study has compared these three methods despite differences in chroma, value, and hue. Therefore, the main aim of the study was to evaluate the difference in the accuracy of tooth shade selection between the conventional visual method, digital photography with a smartphone camera, and the use of a Vita Easyshade spectrophotometer according to shade value, chroma, and hue.

## Materials and methods

Study design

**Figure 1:**
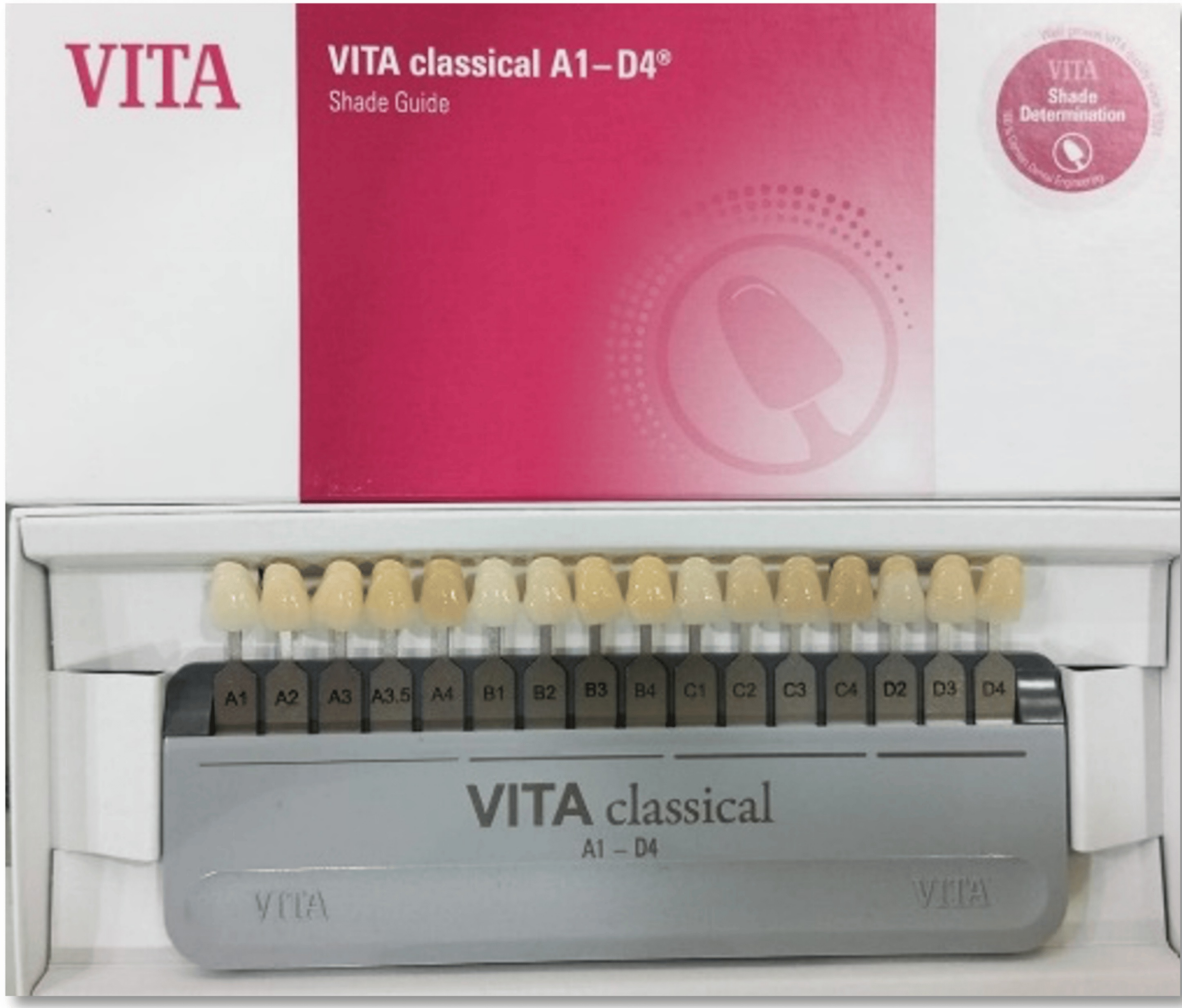
VITA classical shade guide

In the current cross-sectional study, the VITA classical shade guide (VITA Zahnfabrik [Bad Säckingen, Germany]) was used as the study tool to compare the conventional visual method, smartphone camera, and Vita Easy shade (Figure 1). 

Ethical approval was obtained from the Institutional Review Board of Umm al Qura University, Faculty of Dentistry (Approval No. HAPO-02-K-012-2023-11-1868). A consent form was obtained from each participant. Those who declined participation were not included in the study.

Participants

A convenient sample of 82 dental students from Umm Al-Qura University, College of Dental Medicine, in their 5^th^, 6^th^, and 7^th^ years (interns) were selected. 

Visual method

**Figure 2:**
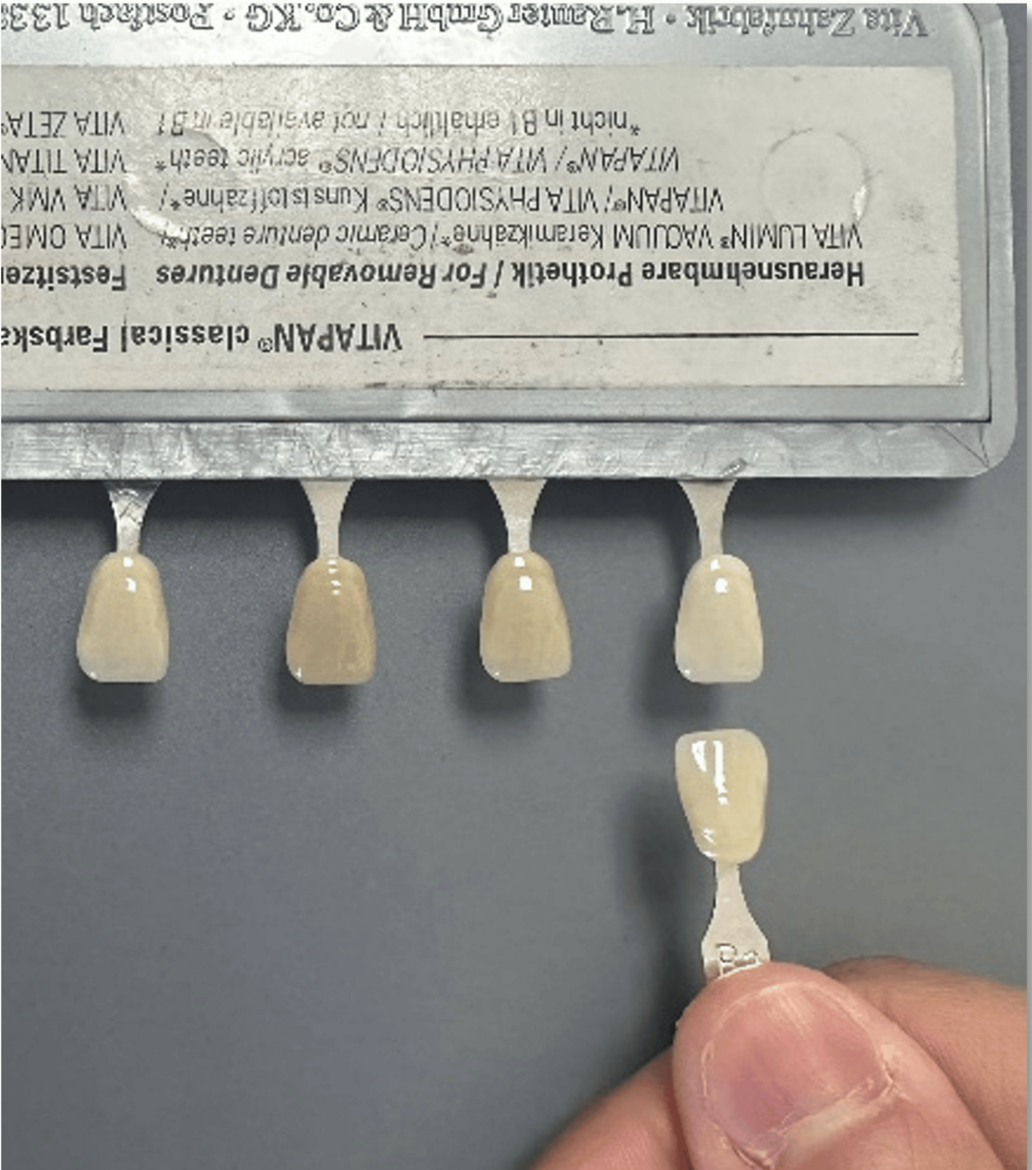
Visual method in shade selection

Study subjects were provided with a B2 shade tab that was taken from the VITA Classical shade guide. Subjects were asked to match the selected B2 shade tab with four other shade tabs arranged based on shade value (A1-B2-D2-A2), chroma (B1-B2-B3-B4), and hue (A2-B2-C2-D2) (Figure 2). Hue is a color description that includes red, purple, blue, etc. Chroma is the intensity or saturation of color, and value is the brightness of the object [14]. The shade category for each shade tab from the VITA shade guide was concealed during the matching process to prevent bias.

Photo method

**Figure 3:**
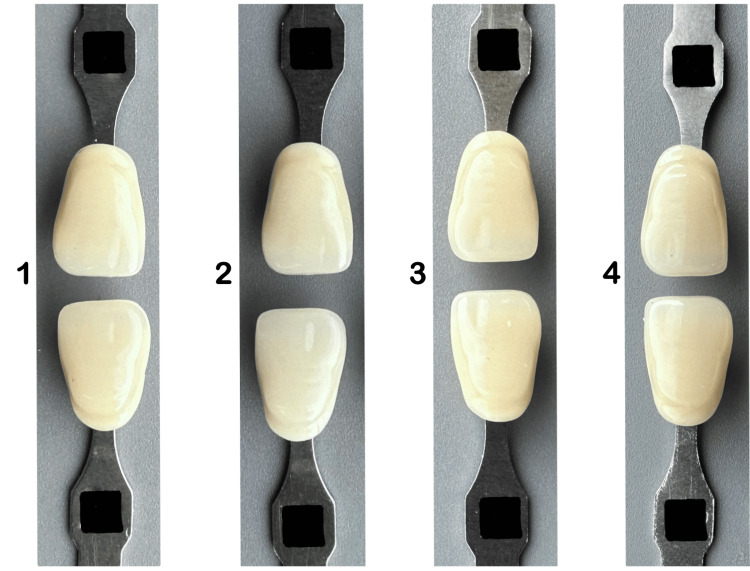
Shade pair arranged randomly according to value (B2-A2, B2-A1, B2-B2, B2-D2), chroma (B1-B2-B3- B4)

**Figure 4:**
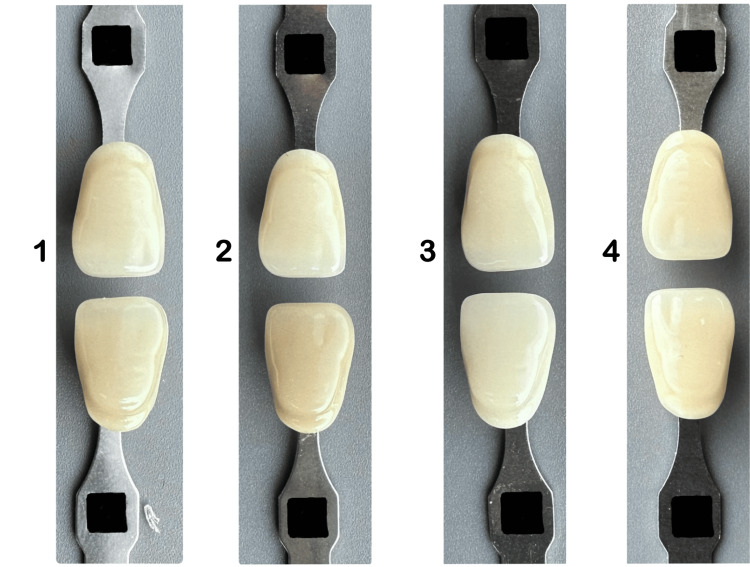
Shade pair arranged randomly according to chroma (B2-B3, B2-B4, B2-B1, B2-B2)

**Figure 5:**
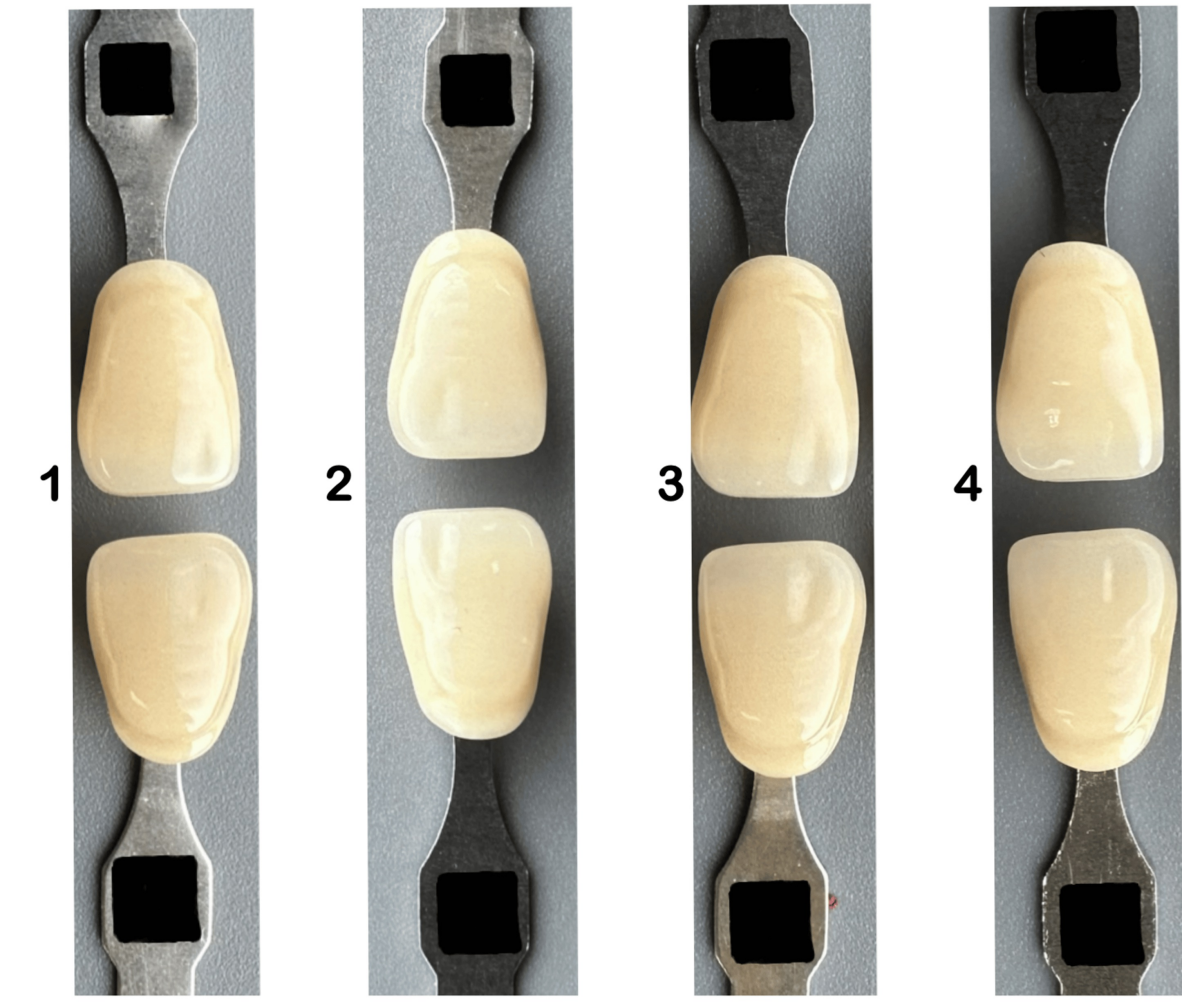
Shade pair arranged randomly according to Hue (B2-A2, B2-B2, B2-C2, B2-D2)

Subsequently, subjects were presented with four images composed of shade pairs in each image that were taken by researchers with an iPhone smartphone camera (13 Pro) under daylight (10 am-12 pm), as shown in Figures 3-5. Then asked the subjects to select the most matched shade pair according to shade value (A1-B2-D2-A2) (Figure 3), chroma(B1-B2-B3-B4) (Figure 4), and hue (A2-B2-C2-D2) (Figure 5).

Also, the names of the shade tabs in the images were concealed to prevent bias.

Easyshade spectrophotometer method

**Figure 6:**
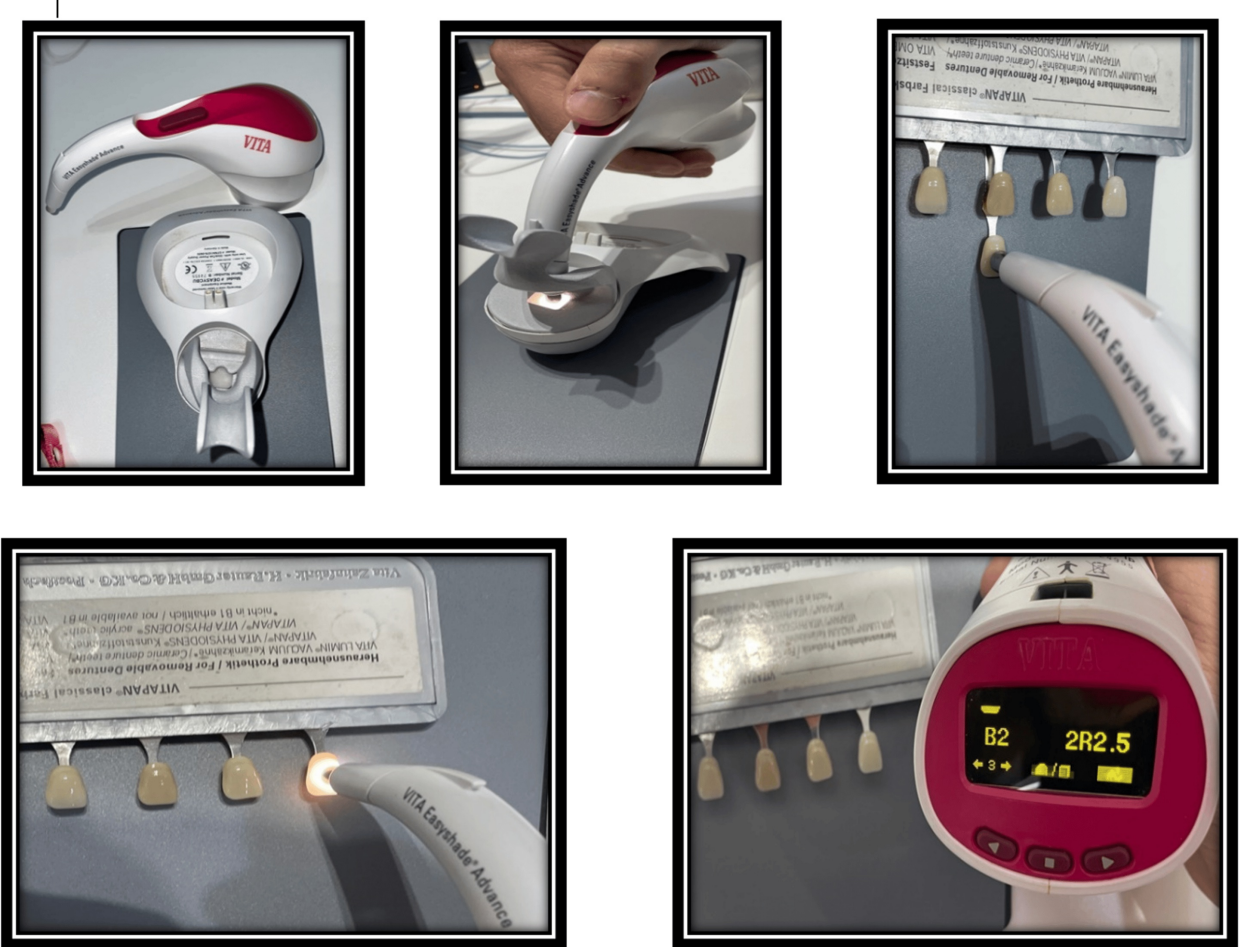
Using a Vita Easyshade spectrophotometer according to the manufacturer's instructions

Next, each participant used a Vita Easyshade spectrophotometer (VITA Easyshade V, Germany) with a gray card as a background to match the shade B2 with other shade tabs arranged based on shade value (A1-B2-D2-A2), chroma (B1-B2-B3-B4), and hue (A2-B2-C2-D2). The spectrophotometer was operated according to the manufacturer's instructions, which are first to place the handpiece in the calibration block holder and press the measurement switch to activate the calibration procedure. Secondly, the probe tip must be placed perpendicular and flush to the tooth surface. Thirdly, steady hold of the probe tip against the center of the tooth, pressing the measurement button and holding the probe tip against the tooth until two rapid "beeps" can be heard to indicate completion of the measurement (Figure 6).

Accuracy was measured. When there was a match between the selected shade from the participant and the used one by the investigator, then it was recorded as correct identification. Otherwise, it was an inaccurate identification.

Statistical analysis

The frequency with a binomial exact 95% confidence interval (CI) for the correct percentage of shade matching was calculated for each method for shade selection. The visual method was used as a reference and compared with both the photo method and Easyshade. The chi-square test or Fisher’s exact if the expected cell frequency was less than five was used to compare the shade selection method and also to compare between males and females. Stata 14 (Stata Corp., USA) was used with a significance threshold of 0.05.

## Results

The current study included 82 subjects, 42 females and 40 males. All results for value, chroma, and hue were elaborated on in Tables 1-3.

**Table 1:** Comparison of value accuracy in shade selection between visual, smartphone camera, and Vita Easyshade Spectrophotometer among dental students in Umm Al-Qura University (p-values were generated from chi-square tests)

Value	Females (n=42). Frequency (%;95% Confidence Interval)	Males (n=40). Frequency (%;95% Confidence Interval)	Total (n=82). Frequency (%;95% Confidence Interval)	p-value
Visual	21 (50.00%: 34.19, 65.80%)	19 (47.50%: 31.51, 63.87%)	40 (48.78%: 37.58, 60.07)	Reference
p-value	0.821			
Photos	25 (59.52%: 43.28, 74.34%)	24(60.00%: 43.33, 75.13%)	48 (58.54%: 47.12, 69.31)	0.965
p-value	0.965			
Easyshade	20 (47.62%: 32.00, 63.58%)	15 (37.50%: 22.73, 54.20%)	35 (42.68%, 31.81, 54.09)	0.002
p-value	0.354			

**Table 2:** Comparison of chroma accuracy in shade selection between visual, Smartphone Camera and Vita Easyshade Spectrophotometer among dental students in Umm Al-Qura University.( P-values were generated from C chi-square or F Fisher exact tests)

Chroma	Females (n=42). Frequency (%;95% Confidence Interval)	Males (n=40). Frequency (%;95% Confidence Interval)	Total (N=82). Frequency (%;95% Confidence Interval)	p-value
Visual	34 (80.95%: 65.88, 91.40%)	37 (92.50%: 79.61, 98.42%)	71 (86.59%: 77.26, 93.10)	Reference
p-value	0.195^F^			
Photos	37 (88.10%: 74.37, 96.02%)	39 (97.50%: 86.84, 99.94%)	76 (92.68%: 84.75, 97.20)	0.094^C^
p-value	0.202^F^			
Easyshade	39 (92.86%: 80.52, 98.50%)	33 (82.50%: 67.22, 92.66%)	72 (87.80%: 78.71, 93.00)	0.674^F^
p-value	0.189^F^			

**Table 3:** Comparison of hue accuracy in shade selection between visual, smartphone camera, and Vita Easyshade Spectrophotometer among dental students in Umm Al-Qura University (p-values were generated from Chi-square or Fisher exact tests)

Hue	Females (n=42). Frequency (%;95% Confidence Interval)	Males (n=40). Frequency (%;95% Confidence Interval)	Total (82). Frequency (%;95% Confidence Interval)	p-value
Visual	7 (16.67%: 6.97, 31.36%)	12 (30.00%: 16.56, 46.53%)	19 (23.17%: 23.17, 14.56)	Reference
p-value	0.153^ C^			
Photos	28 (66.67%: 50.45, 80.43%)	16 (40.00%: 24.86, 56.67%)	44 (53.66%: 42.29, 64.75)	0.094^C^
p-value	0.015^C^			
Easyshade	5 (11.90%: 3.98, 25.63%)	3 (7.50%: 1.57, 20.39%)	8 (9.76%: 4.31, 18.32)	0.674^F^
p-value	0.713^F^			

**Figure 7:**
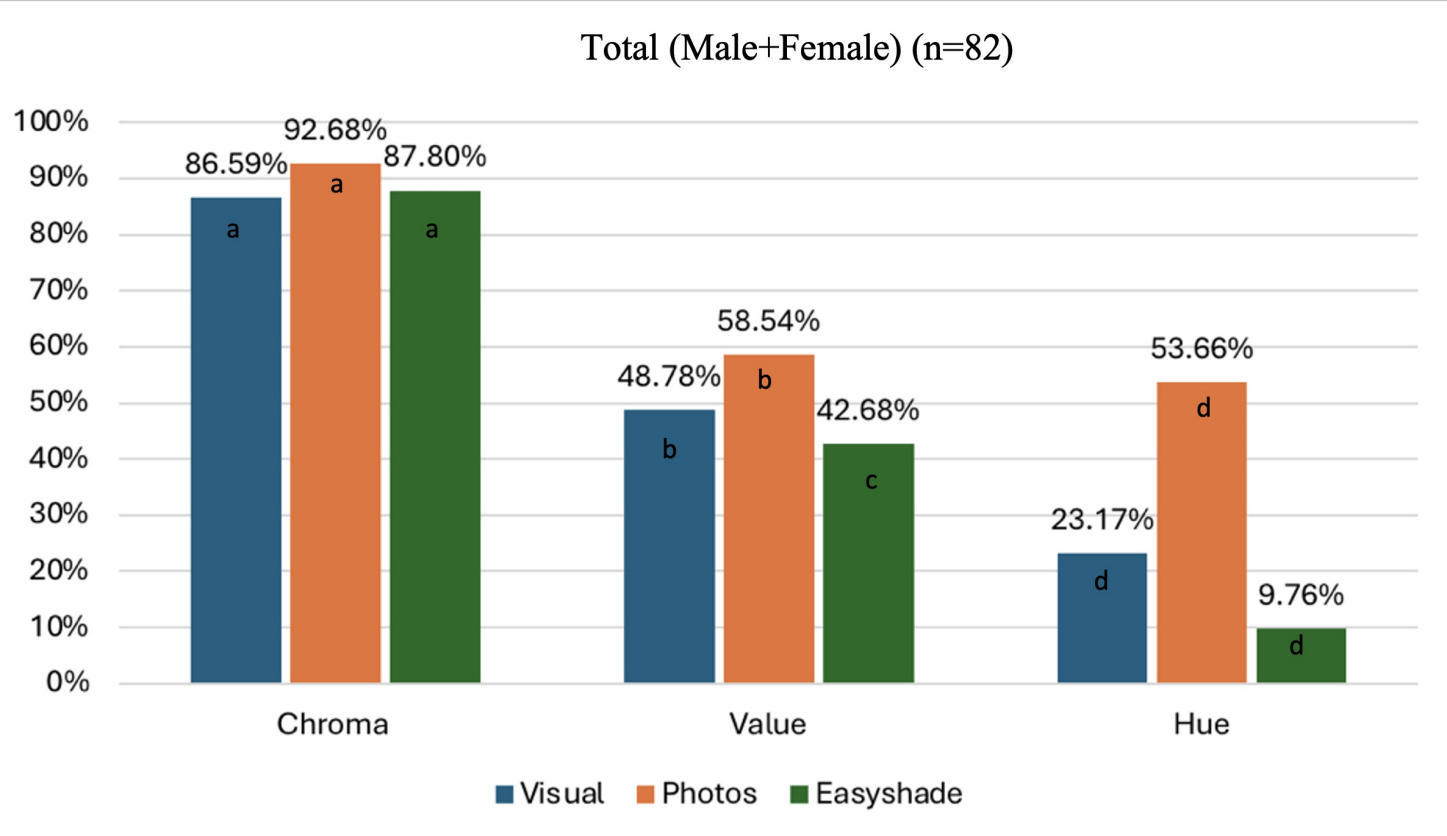
Comparison of the accuracy in shade selection between Visual, Smartphone Camera, and Vita Easyshade Spectrophotometer among dental students in Umm Al-Qura University. Different letters denote a significant difference compared to the reference group (visual) (p < 0.05)

There was no significant difference between male and female students in the measured accuracy in shade selection using the three methods, except for hue when the photos method was used (p=0.015). For value (p=0.965), chroma (p=0.094), and hue (p=0.094), the photo method was comparable in accuracy to the visual method. Although the accuracy was higher for the photo method, it was not statistically significant. The Easyshade was also comparable to the visual method in chroma (p=0.674), statistically significantly lower when assessing value (p=0.002), and not statistically significant when assessing hue (p=0.094). Comparing the results of the same method, the accuracy of shade selection was highest for chroma, followed by value, and lowest for hue (Figure 7). 

## Discussion

Given that one of the essential elements of successful dental treatment is esthetic accuracy, achieving an optimal tooth shade match is crucial for the success of cosmetic procedures [13]. Various tools and approaches are available to help dentists make decisions in shade selection. In our study, we compared the accuracy of three methods: the conventional visual technique, digital photography with a smartphone camera, and the Vita Easyshade spectrophotometer. Our findings indicated that digital photography with a smartphone camera exhibited the highest accuracy in chroma matching (92.68%), compared to the conventional visual method (86.59%) and the Vita Easyshade spectrophotometer (87.80%) (Figure 7). Similarly, in value selection, digital photography with a smartphone camera demonstrated superior accuracy (58.54%) relative to the conventional visual method (48.78%) and the Vita Easyshade spectrophotometer (42.68%) (Figure 7). In hue matching, digital photography with a smartphone camera also performed better (53.66%) than the conventional visual method (23.17%) and the Vita Easyshade spectrophotometer (9.76%) (Figure 7).

Utilizing smartphones for shade selection in dentistry has demonstrated promising results in enhancing accuracy and efficiency, suggesting they could be a reliable tool for shade matching. Several factors contribute to the improved outcomes compared to other methods. Firstly, the standardization of the selection method plays a critical role. Secondly, the consistency of the shooting angle is maintained. Thirdly, the uniformity of the lighting angle and intensity, which was standardized to daylight (10 a.m. to 12 p.m.), contributes to the reliability of the results.

According to a systematic review, smartphones with digital shade-matching applications can analyze and match tooth shades more consistently than traditional visual methods [13]. These applications leverage the phone’s camera to capture high-resolution images and apply color correction, thus minimizing subjective errors. This technology is not only cost-effective but also facilitates enhanced communication with dental laboratories by enabling the easy sharing of precise shade information, which leads to improved clinical outcomes [15]. However, light reflection, or specular reflection, can cause glare on photographs taken with smartphones, obscuring important color details needed for accurate shade selection. This reflection can create a glossy appearance on the tooth surface, which may hide or distort the true color, leading to less accurate color matches [16].

A previous study showed that the visual method of shade selection is comparable to digital photography, particularly when conducted under controlled lighting conditions [17]. This correlation aligns with the findings of our study. Moreover, our findings reveal that digital photography using a mobile phone camera exhibits higher accuracy in identifying hue compared to the visual method. This advantage could be attributed to image processing algorithms, which enhance color detection and reproduction. 

Additionally, it is important to consider that other studies consistently identify the spectrophotometer as the most accurate tool for shade selection [12]. In general, spectrophotometers have multiple drawbacks: they are high cost and expensive, require a certain level of expertise to operate, are sensitive to changes in environmental conditions like temperature, humidity, and ambient light, and only measure color, not surface texture [18]. Our research yielded different results: the spectrophotometer showed the lowest matching percentage among the methods tested. This discrepancy could be due to several factors specific to our unit that affected its long-term performance. For instance, shade calibration issues, even when following the exact manufacturer instructions, led to discrepancies in some readings, which might have affected the accuracy. Additionally, a battery charge that is not at the optimal level might have compromised the device’s performance, leading to inconsistent results. This is critical for maintaining high standards in dental practice. Shade calibration problems were found to be common in dental practice [19]. Proper calibration is essential for accurate shade determination. These potential issues highlight the importance of ensuring optimal operating conditions and regular maintenance for spectrophotometers to achieve the highest level of accuracy.

The future of digital shade matching in dentistry appears promising, thanks to advancements in artificial intelligence, which are expected to enhance the accuracy and efficiency of the process [13]. The ongoing growth in the use of smartphone applications for shade matching will likely continue, making this technology more accessible to a wider range of dental professionals. These apps can be regularly updated with new features and improvements, offering cost-effective solutions for dental practices around the world [19]. Additionally, digital shade-matching tools will improve communication between dental clinics and laboratories by enabling the precise sharing of shade information, leading to better coordination and restorations that more accurately match the patient’s natural teeth [20].

This study has its limitations, underscoring the necessity of considering multiple factors and methodological nuances when comparing different tools for color selection, ultimately contributing to a more comprehensive understanding. Specifically, the study could be improved by adding multiple smartphone types and digital cameras when using the three methods for shade selection. Additionally, students have less experience compared to graduate dentists, which may affect their ability to select the right shade. Another investigation involving dentists and specialists could be beneficial. Moreover, the study did not perform a sample size calculation and used only a convenient sample of 82 participants, which could be increased for better reliability. Additionally, each participant was interviewed once. Conducting a longitudinal study with multiple interviews per participant could enhance the findings. In our study, we used only the VITA Classical shade guide. Future studies could incorporate the 3D Master shade guide. We did not use flash for illumination, relying instead on sunlight for standardization. Future studies should include a greater number of shade tabs in the comparison process, rather than limiting it to four, to obtain more comprehensive results. Due to budget constraints, we used a single Easyshade spectrophotometer. Future studies could benefit from using multiple devices to enhance reliability.

## Conclusions

Within the limitations of this study, digital photography using a smartphone camera has shown promising outcomes as a method for shade selection that clinicians could consider in their practice. This technique not only demonstrates accuracy in matching chroma, value, and hue but also offers a cost-effective and efficient option for enhancing aesthetic outcomes in dental practices. The standardization of the selection process, consistency in shooting angles, and uniform lighting conditions play a significant role in its reliability. Despite challenges when using this method, digital photography with smartphones could improve the precision of shade matching and communication with dental laboratories.
